# Supplementary material for: Content-rich biological network constructed by mining PubMed abstracts
Source: BMC Bioinformatics. 2004 Oct 8;5:147. doi: 10.1186/1471-2105-5-147 (PMC528731; doi:10.1186/1471-2105-5-147)
Supplement: Additional File 2 — The original results of the above study (non-essential files are deleted to keep the file size under the limit set by BMC bioinformatics). [file 1471-2105-5-147-S2.bz2 › chilibotAdditionalFile2/dip10/660ID2118521E11051/html/CBBL_CBBS.html]

 


 **CBBL** and **CBBS** 
  
Found 12 abstracts in PubMed, retrieved 10.  
 

 What does Google say? 
 PDF only 
| .edu only 

---

**Interactive relationship** (e.g. stimulation, inhibition, etc)

**Neutral relationship**- The deduced gene products,  **CbbL**  and  **CbbS** , had 93 and 87% identity with Thiobacillus intermedius  **CbbL**  and Nitrobacter winogradskyi  **CbbS** , respectively.  Ref: 12005060 Biosci Biotechnol Biochem, 2002

**Non-interactive relationship** (e.g. studied together, co-existance, homology, etc.)

- Endogenous T. denitrificans promoters were shown to function in R. sphaeroides, resulting in high levels of  **cbbL**   **cbbS**  and cbbM expression in the R. sphaeroides host.  Ref: 8550452 J Bacteriol, 1996
- Analysis of RNA showed that  **cbbL**  and  **cbbS**  are cotranscribed on a message approximately 00 nucleotides in size.  Ref: 9696760 J Bacteriol, 1998
- These studies suggest that the cbbRI  **cbbL**   **cbbS**  cbbQ genes were acquired by Rh.  Ref: 9467914 Microbiology, 1998
- Analysis of the nucleotide sequence of the form I ribulose bisphosphate carboxylase oxygenase RubisCO genes  **cbbL**  and  **cbbS**  of the non sulfur purple bacterium Rhodobacter capsulatus indicated that the deduced amino acid sequence of the large subunit was not closely homologous to the large subunit from related organisms.  Ref: 9467914 Microbiology, 1998
- The deduced primary sequence  **cbbL**  and  **cbbS**  of form I ribulose bisphosphate carboxylase oxygenase rubisco from Bradyrhizobium japonicum places this enzyme within the Type IC subgroup of red like rubisco enzymes.  Ref: 9882445 Arch Biochem Biophys, 1999
- Genes encoding the large subunit  **cbbL** , small subunit  **cbbS**  and putative regulatory gene cbbQ were shown to be located on one cluster.  Ref: 11889481 Arch Microbiol, 2002
- Surprisingly, a cosmid clone containing the R. capsulatus form I RubisCO genes  **cbbL**  and  **cbbS**  failed to hybridize to the other cbb structural gene probes, unlike the situation with the closely related organism R. sphaeroides.  Ref: 8588741 Arch Microbiol, 1995
- Genes coding for both large  **cbbL**  and small  **cbbS**  subunits of a RubisCO enzyme were identified, isolated and sequenced.  Ref: 8828223 Microbiology, 1996
- Expression of  **cbbL**  and  **cbbS**  in Escherichia coli led to the detection of RubisCO activity in the presence of 0.1 mM isopropyl beta D thiogalactopyranoside IPTG.  Ref: 12005060 Biosci Biotechnol Biochem, 2002
- Isolation and characterization of  **cbbL**  and  **cbbS**  genes encoding form I ribulose bisphosphate carboxylase oxygenase large and small subunits in Nitrosomonas sp.  Ref: 12005060 Biosci Biotechnol Biochem, 2002
- In the present study,  **cbbL**  and  **cbbS**  were cloned and sequenced.  Ref: 9696760 J Bacteriol, 1998
- Four genes encoding carboxysome shell peptides csoS1A, csoS1B, csoS1C, csoS2, the genes encoding the large and small subunits of RuBisCO  **cbbL** ,  **cbbS** , and three unidentified ORFs constitute an operon in Thiobacillus neapolitanus.  Ref: 10816046 Arch Microbiol, 2000
- The  **cbbL**   **cbbS**  and cbbM genes of Thiobacillus denitrificans, encoding form I and form II ribulose bisphosphate carboxylase oxygenase RubisCO, respectively, were found to complement a RubisCO negative mutant of Rhodobacter sphaeroides to autotrophic growth.  Ref: 8550452 J Bacteriol, 1996
- the genes for the enzyme,  **cbbL**  and  **cbbS** , are part of a putative carboxysome operon.  Ref: 9696760 J Bacteriol, 1998
- The  **cbbL**  and  **cbbS**  genes encoding form I ribulose bisphosphate carboxylase oxygenase RubisCO large and small subunits in the ammonia oxidizing bacterium Nitrosomonas sp.  Ref: 12005060 Biosci Biotechnol Biochem, 2002
